# The Significance of the Collegiate Year, as a Requirement to Medical Matriculation

**Published:** 1913-02

**Authors:** 


### BUFFALO MEDICAL JOURNAL

**A Monthly Review of Medicine and Surgery**

EDITOR AND PUBLISHER DR. A. L. BENEDICT, 228 Summer St., corner of Elmwood Ave., Buffalo. (Address for all communications. Please make personal and telephone calls before 1 P. M.)

THE BUFFALO MEDICAL JOURNAL is the third, in age, in the western continent. It is independent but supports professional organization. As a scientific medium, t is unrestricted in territory. As a news medium. it is limited mainly to the western four districts of the Medical Society of the State of New York. The co-operation of physicians is asked in securing personal, obituary and other news items. Secretaries of Medical Societies in this region are requested to send brief reports of meetings. Full transactions will be published at cost of composition.

Subscription $2.00 a year in advance; $3.00 for two years in advance. Changes and errors in address or failure or duplication of delivery, should be reported immediately, Back numbers will be supplied if possible but requests for copies should be made promptly,

YEARLY VOLUME 68. FEBRUARY, 1913 No. 7

*"Beauty is only skin deep" originated with a man who had never seen a Haversian system, a section of the retina or a blood mount.*

### The Significance of the Collegiate Year, as a Requirement to Medical Matriculation

At first thought, it would appear that the requirement of one more year of preliminary education for medical students is of minor importance, and that barring the slight mortality at the corresponding age and the chances of diversion of interests by accidental causes, as many students would enter medical colleges as formerly. We are inclined to prophesy, however, a very radical change from this further entrance of the wedge.

First of all, universities in the sense of educational institutions combining collegiate and professional courses, will be given an enormous advantage from the tendency of students generally to preserve an established affiliation or, at least, to pass from one to another institution of essentially the same grade and customs. This tendency involves an injustice to many worthy independent colleges and medical schools, but it cannot be denied that, on the whole, it is in line with the general tendency to the survival of the fittest. The better class of independent schools will probably affiliate in such a way as to secure both material strength and facilities for improved instruction. Since many universities exist with departments in two towns, it is obvious that combinations of independent schools need not involve the removal of either.

Moreover, while it is not true that every independent college and professional school is either weak or ethically objectionable, it is true in the aggregate that the best class of schools of all kinds are parts of universities and that the undesirable institutions are mainly independent organizations. Thus, solely from the standpoint of thoroughness, the additional requirement for matriculation has a much more radical tendency than appears at first sight, since it will almost inevitably place an increasing number of students under a stricter supervision.

It needs no argument to show that a requirement which is not only quantitatively greater but qualitatively more stringent will have a marked influence in diminishing the number of matriculants. But the mere fact of passing from the full high school standard to the beginning of the college standard will probably have a far greater influence in this direction. Without in any sense exaggerating the importance of college training—and we personally incline to the view that it has been much exaggerated in the past—it must be admitted that the horizon of the college freshman, so far as scholastic and professional matters are concerned, is much broader than that of the high school senior. Once settled in college, almost every influence is at work to retain the student there up to the completion of the course, except a regard for financial expense and the expense in time invested. Fraternities dislike to accept a member who will leave at the end of a year. Many of the more desirable athletic and other college organizations have the same rule, or do not even admit to membership till after the first year. The influence of faculty, fellow students and often the student's family and the intrinsic charm of college life, as well as awakened ambition, are all strongly for a full course or, at least, for the standard combination college and professional course of six or seven years.

Again, there is the feeling that with only one year to be saved from the approved combination course of the better grade universities, it does not pay to stop short with the minimum requirement, and there is also a strong temptation on the part of one taking the six-year combination course to add another year and secure both the professional and the baccalaureat degree. Thus, we are inclined to think that the legal requirement of one year in college will induce fully half of all prospective medical students to take at least two years. It is obvious, therefore that, if the premises are correct, the increased requirement will have a much greater economic, restrictive effect on medical matriculation, than appears on the surface.

But this is not all. The ambitions, judgment and selective affinities of a youth who has decided to study medicine and who has, for that purpose, rather reluctantly or at least perfunctorily, finished a high school course or "made up points," are very different from those of the same youth, a year or two older and well started on a college course. He is now one of the (scholastically) upper 2½ per cent. instead of the upper 10 or 12 per cent. Except for his tentative choice of a profession, his future is not limited to this any more than is that of his classmates. Several professions and various lucrative business careers are equally within his grasp and, in the natural course of events, many boys who have gone to college expressly to conform to the medical matriculation law, will consider themselves better adapted to other callings.

We are inclined, therefore, to regard the increase in matriculation standards, comparatively insignificant as it first appears, as of the utmost significance, first in helping to solve certain ethical problems regarding medical education and, secondly, by limiting to a considerable degree the supply of physicians and hence tending to restore the equilibrium between supply and demand. And this second influence is perhaps more important because a sound economic basis is the greatest prophylactic of ethical failures in any profession or business.

